# The Clinical Guiding Role of the Distribution of Corneal Nerves in the Selection of Incision for Penetrating Corneal Surgery in Canines

**DOI:** 10.3390/vetsci8120313

**Published:** 2021-12-08

**Authors:** Zichen Liu, Chang Yu, Yiwen Song, Mo Pang, Yipeng Jin

**Affiliations:** College of Veterinary Medicine, People’s Republic of China Agricultural University, No. 2 Yuanmingyuan West Rd, Haidian District, Beijing 100193, China; lzc94@126.com (Z.L.); yuchangandy@gmail.com (C.Y.); vetsongyiwen@163.com (Y.S.); pangmo1991@126.com (M.P.)

**Keywords:** canine, corneal nerve staining, gold chloride, corneal incision

## Abstract

The cornea is one of the regions with the highest density of nerve terminals in the animal body and it bears such functions as nourishing the cornea and maintaining corneal sensation. In veterinary clinical practice, the corneoscleral limbus incision is frequently applied in cataract surgery, peripheral iridectomy, and other procedures for glaucoma. Inevitably, it would cause damage to the nerve roots that enter the cornea from the corneal limbus, thus inducing a series of complications. In this paper, the in vitro cornea (39 corneas from 23 canines, with ages ranging from 8 months old to 3 years old, including 12 male canines and 11 female canines) was divided into 6 zones, and the whole cornea was stained with gold chloride. After staining, corneal nerves formed neural networks at different levels of cornea. There was no significant difference in the number of nerve roots at the corneoscleral limbus between different zones (F = 1.983, *p* = 0.082), and the nerve roots at the corneoscleral limbus (mean value, 24.43; 95% CI, 23.43–25.42) were evenly distributed. Additionally, there was no significant difference in the number of corneal nerve roots between male and female canines (*p* = 0.143). There was also no significant difference in the number of corneal nerve roots between adult canines and puppies (*p* = 0.324). The results of the above analysis will provide a reasonable anatomical basis for selecting the incision location and orientation of penetrating surgery for the canine cornea in veterinary practice.

## 1. Introduction

The corneal surface is one of the zones with the highest density of nerve terminals, in which the density of corneal epithelial nerve terminals is 20–40 times that of nerve terminals in the dental pulp and 300–600 times that of cutaneous epithelial nerve terminals [1,2]. There are numerous sensory nerves and pain receptors on the corneal surface, which are mainly innervated by the long ciliary nerves derived from the ophthalmic branch of trigeminal nerves. Among them, the primary pain receptors are located in the epithelial layer, while the pressoreceptors are mainly located in the stromal layer [3].

Corneal nerves not only possess the sensory function, but also provide various metabolic nutrition and support for the cornea, including regulating ion transport, the proliferation, differentiation, adhesion, and migration of cells and wound healing. Therefore, they are significant for maintaining the normal structure and function of the cornea [4]. After corneal nerves are injured by various corneal operations, not only will the corneal sensation decrease, but also the corneal nutrition and metabolic disorders of different degrees may occur consequently [5,6]. The corneal nerve dysfunction can induce reduced mitosis of corneal epithelial cells, decreased adhesion activity between cells, decreased wound healing ability, and secondary neurotrophic corneal epithelial diseases, which are manifested as xerophthalmia, corneal infection, corneal ulcer, and so forth [7]. Corneal penetrating surgery would cause the most serious damage to corneal nerves. After the penetrating incision is performed on the cornea at the corneoscleral limbus, corneal nerve trunk would be cut off, and the cornea near the incision would be free from the innervation, which makes the sensation become dull or disappear. The corneal sensation in corresponding zones would recover slowly. In animal experiments, it can be found that after the penetrating incision is performed on the corneal limbus of rabbits for 1–3 months, stromal corneal nerves could penetrate scar tissue and re-enter the corresponding parts of the cornea; however, the subepithelial nerve plexus and interepithelial nerve terminals are still missing or scarce 30 months after the operation [8,9,10]. After the cornea is stimulated by penetrating surgery, nerve terminals will release some neurotransmitters, including acetylcholine (ACE), catecholamine (CA), substance P (SP), and calcitonin gene-related peptide (CGRP), which make the sensitivity of the corneal 100 times that of the conjunctiva. Corneal lesions (such as keratitis, corneal foreign bodies, corneal ulcers, etc.) in any depth can induce severe pain and blepharospasm [11,12,13].

The canine cataract is a significant ophthalmic disease in clinic. Surgery is the only effective method in the treatment of canine cataract. The transparent corneal incision or scleral tunnel incision is usually applied in the process of cataract surgery. Currently, the transparent corneal incision is often performed in the direction of 12 o’clock in pet clinics [14]. It is prone to the development of postoperative complications, such as corneal edema, delayed corneal epithelial healing, corneal endothelial dystrophy, decreased corneal perception, and decreased intraocular pressure [15,16,17]. Partial symptoms are closely related to corneal nerve injury [18,19,20]. Therefore, the occurrence of postoperative complications can be effectively reduced via exploring the difference of nerve distribution in different corneal positions of canines and selecting the zone with relatively few corneal nerve roots as the incision position. Furthermore, the corneal nerve distribution can provide a reasonable anatomical basis for the selection of incision position during the corneal penetrating surgery in veterinary clinical practice.

Currently, most understanding about corneal nerve morphology and distribution is obtained by corneal nerve staining, including gold chloride staining, acetylcholinesterase staining, and immunohistochemical staining [21]. Gold chloride corneal nerve staining is a specific staining method for nerve terminals. There are various methods to stain corneal nerves with gold chloride reported in the literature [22,23,24,25]. The principle of gold chloride staining is generally considered as that gold ions in solution are reduced into gold particles in an acidic environment, which would attach to neurofibrils for color manifestation. The silver nitrate staining, gold chloride staining, and cholinesterase staining have been subjected to a comparison in the literature [26]. It has been concluded that gold chloride staining can clearly manifest nerve fiber bundles and subepithelial nerve plexus in corneal parenchyma, as well as the extremely tenuous nerve fibers in corneal parenchyma [27]. Besides, gold chloride staining can manifest corneal nerves in every layer of the cornea, especially the nerve terminals in the epithelial layer and stromal layer. However, gold chloride staining is limited by its unfavorable specificity, which induces failure in the discrimination of various corneal nerves. The objective of this study was to analyze the distribution patterns of corneal nerve roots at different locations. In order to avoid wrong or missing nerve roots, the gold chloride staining method with poor specificity was adopted, due to the fact that this method can stain nerves in the stromal layer and epithelial layer and manifest the extremely tenuous nerve fibers.

## 2. Materials and Methods

### 2.1. Animal and Tissue Sample Collection

All canines (mixed breed canines) in this experiment were of medium size and they died of non-ophthalmic diseases, with ages ranging from 8 months old to 3 years old. Among them, 9 canines were less than 1 year old, 8 canines were between 1–2 years old, and 6 canines were between 2–3 years old. The death causes of canines were mainly caused by congenital diseases and trauma. The eyeballs were collected within 1 h after the canine died. Afterwards, they were placed in fresh 4% formaldehyde solution for later use; 39 corneas from 23 canines were successfully stained, including 12 male canines and 11 female canines (21 corneas from the left eyeballs and 18 corneas from the right eyeballs).

**Figure 1:**
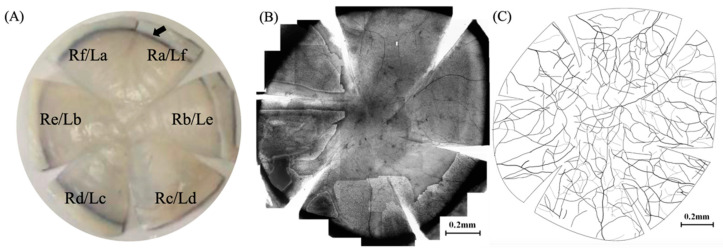
Before corneal staining, (**A**) the positions of the six zones (Ra–Rf, La–Lf) made on the isolated canine cornea. The black arrow indicates the corneoscleral limbus. Photoshop software was used to stitch (**B**) the cornea staining maps in each zone and draw (**C**) the nerve direction.

Eyeballs were collected within 1 h after the death of canines, and were marked in the direction of 12 o’clock before completely removing them. After taking out these eyeballs, the blood was flushed with normal saline first. Subsequently, these eyeballs were dried with the gauze and placed into a jar containing formalin solution (4% formaldehyde solution). Then, the cornea was removed from the fixed eyeball along the corneoscleral limbus, and a short incision was made with ophthalmic scissors in the direction of 12 o’clock for marking. The corneal endodermis and Descemet’s membrane were torn with toothed forceps. Long incisions were made in the direction of 2 o’clock, 4 o’clock, 6 o’clock, 8 o’clock, and 10 o’clock, respectively, so that the cornea was divided into 6 quadrants. In view of the symmetry of both eyeballs, 6 corneal zones of the right eyeball of canine were marked as Ra, Rb, Rc, Rd, Re, and Rf, respectively, in a clockwise direction, while 6 corneal zones of the left eyeball were marked as La, Lb, Lc, Ld, Le, and Lf, in a counterclockwise direction (Figure 1A). Five researchers were invited to independently count the number of nerve roots entering the cornea from the corneal limbus, and finally conduct statistics and analysis.

### 2.2. In Vitro Cornea Staining

The cornea was washed three times with 0.1 M PBS buffer and placed in acetic acid solution for 15 min. After being washed with distilled water, the cornea was stained in 1% gold chloride solution away from light for 15 min. After being washed with distilled water, the cornea was placed in acetic acid solution, and after the incubation for 6 h, the corneal staining was observed under a microscope. The cornea was taken out under an appropriate condition (the nerves are clearly stained and there is no excessive background staining). The cornea was immersed and washed in 0.1 M PBS buffer solution for 10 min, then placed in fixing solution (containing sodium thiosulfate, sodium sulfite, acetic acid, and aluminium alums) and treated away from light for 10 min. The cornea was taken out from the fixing solution, and the residual liquid was washed away with 0.1M PBS buffer. Then, the cornea was stored in 0.1M buffer. Three drops of PBS buffer were added on the slide, and the cornea was laid flat on the slide. The cornea was pressed with another slide, and both ends of slides were fixed with fishtail clips. Subsequently, the observation was performed with a binocular optical microscope (Leica), which was connected to a computer for photo obtainment [27,28,29].

### 2.3. Statistical Analysis

The statistical analysis software SPSS Statistics v20 (IBM, New York, NY, USA) was adopted for statistics and data processing, and one-way analysis of variance (ANOVA) was adopted to calculate the population mean interval based on t-distribution. *p* < 0.05 indicates that there is a significant difference, and *p* < 0.01 indicates that there is an extremely significant difference. One-way ANOVA was adopted to test whether there were significant differences in the number of corneal limbus nerve roots among different zones. Besides, Tukey’s method and the SNK-q test were adopted to compare the mean number of nerve roots at the corneoscleral limbus in each corneal zone.

## 3. Results

### 3.1. Corneal Nerve Staining

**Figure 2:**
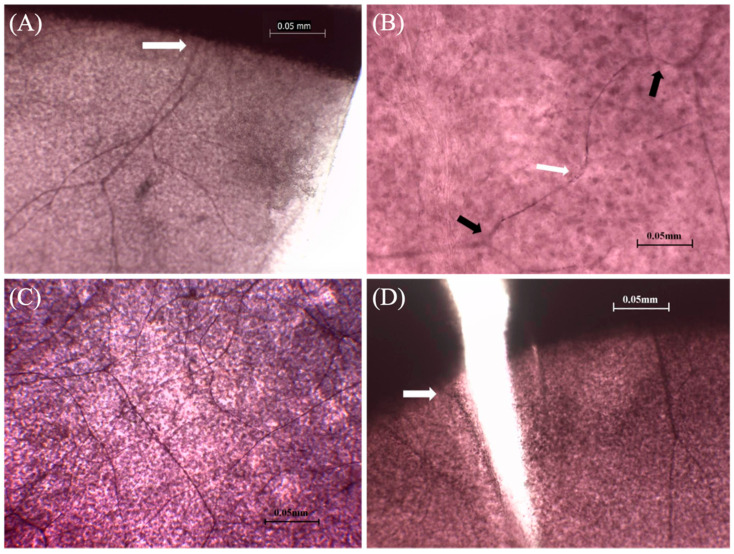
It was observed by optical microscope (40×) that the nerve roots (**A**) (white arrow) entered the cornea from the corneoscleral limbus, (**B**) extended to the center of the cornea, gradually branched (white arrow) and anastomosed (black arrow), and formed the corneal neural network (**C**) at different levels of the cornea. (**D**) Nerve roots (white arrow) located near the corneal incision made before corneal staining.

As per the staining results, the nerve fibers of corneal nerve roots of canines emanated from the corneoscleral limbus to the central cornea (Figure 2A), gradually branched and intersected after entering the cornea (Figure 2B), and formed corneal neural networks at different layers of corneas (Figure 2C). Photoshop software (Adobe Photoshop cc 2015) was adopted for processing and splicing (Figure 1B), and the venation map of canine corneal nerve fiber distribution is presented (Figure 1C). When the nerve root is located at the incision (Figure 2D) in the statistical process, the nerve root is recorded in the latter zone. The average value of the number of nerve roots at the corneoscleral limbus of canines was 24.43, the 5% revised average value was 24.29, and the 95% confidence interval (95% CI) was 23.43–25.42 (Table 1).

### 3.2. Differences in the Number of Nerve Roots at the Corneoscleral Limbus between Male and Female Canines

One-way ANOVA was adopted to analyze the impact of different genders on the number of nerve roots at the corneoscleral limbus. The average number of nerve roots at the corneoscleral limbus in male canines was 24.94 (95% CI, 23.49–26.40), while that in female canines was 23.82 (95% CI, 22.38–25.26) (Table 2). The F value of test statistic between male and female canines was 2.157. The degree of freedom of samples between groups was 1 and the *p* value was 0.143. It could be found that there was no significant statistical difference in the number of nerve roots at the corneoscleral limbus between male and female canines (Table 3).

### 3.3. Distribution of Nerve Roots at the Corneoscleral Limbus of Oculus Sinister and Oculus Dexter

The average number of nerve roots at the corneoscleral limbus in OS was 24.56 (95% CI, 23.19–25.93), while that in OD was 24.27 (95% CI, 22.66–25.87) (Table 4). The F value of test statistics between OS and OD samples was 0.148. The degree of freedom of samples between groups was 1 and the *p* value was 0.700. It can be verified that there was no significant difference in the distribution patterns of nerve roots between the OS and OD (Table 5).

### 3.4. Distribution of Nerve Roots at the Corneoscleral Limbus in Different Zones

One-way ANOVA was adopted to test the difference in the number of nerve roots at the corneoscleral limbus among corneal zones a, b, c, d, e, and f (Table 6). The F value test statistics between groups was 1.993, and the significance level of data was 8.1%, which was higher than the confidence level of 5%. Therefore, there was no significant difference in the distribution of the number of nerve roots at the corneoscleral limbus among six zones. Tukey’s method was adopted to compare and analyze the data of a, b, c, d, e, and f zones, and the statistical difference of the number of nerve roots at the corneoscleral limbus among each zone was tested. It can be seen that the significant difference of the number of nerve roots at the corneoscleral limbus in each zone of the sample was above 10% compared with that in other zones, which was much greater than the confidence level of 5% (Table 7).

### 3.5. Analysis of the Number of Nerve Roots in Corneoscleral Limbus in Different Age Canines

In this experiment, there were 9 canines less than 1 year old, 8 canines between 1–2 years old, and 6 canines between 2–3 years old. The F value of test statistic between corneal samples of canines of different ages was 1.192. The degree of freedom of samples between groups was 2 and the *p* value was 0.324. The analysis of variance showed that there was no significant difference in the number of corneoscleral limbus nerve roots among the three age groups (Table 8).

## 4. Discussion

In this study, it was found that the number of corneal nerve roots had no correlation with gender and age of canines. However, He et al. [26] compared the number of human corneal nerve roots at different ages, in 2010. In the eye samples of donors aged 70 and over, it was found that the corneal nerve density decreased gradually with the increase of age. The author believes that aging reduces the number of central epithelial nerve terminals. In our study, the samples were mainly concentrated in canines aged from 8 months old to 3 years old. It is generally believed that canines under 1 year old are puppies and canines from 1 to 7 years old are adults. The results of this experiment show that there is no significant difference in the number of corneal nerve roots between adult canines and puppies. However, due to the limited age span of the samples, no corneal samples of elderly canines (>7 years old) were collected; thus, it is impossible to analyze the difference in the number of corneal nerve roots in aging canines. However, for the current sample, we believe that the number of corneal nerve roots will not change greatly during the transition from infancy to adulthood. At the same time, He et al. [26] found that epithelial nerve density and terminal numbers were higher in the center of the cornea, rather than the periphery. In our study, it was found that although the distribution density of nerve terminals at the corneoscleral limbus of canines was low, it was the area of nerve root distribution. From this area, nerve terminals were sent out and interwoven in the center of cornea to form a high-density corneal nerve terminals network. This finding is consistent with the results of He et al. [26].

In addition, this paper adopts a six-quadrant zoning method for cornea, which is different from the four-quadrant zoning method proposed by Weigt et al. [23] and He et al. [26]. During the staining process, it could be observed that the staining quality around the incision in the direction of 12 o’clock for marking was higher than that in the contralateral side. Therefore, a short incision (1/4 radius length) was made along the radius in the direction of 12 o’clock when performing the initial marking in this direction, while long incisions (3/4 radius length) were made along the radius in the direction of 2 o’clock, 4 o’clock, 6 o’clock, 8 o’clock, and 10 o’clock, respectively, so that the cornea could be divided equally into six quadrants for the optimal staining results.

The canine corneal diameter is 14–18 mm, the average number of nerve roots is 24.43, and the average density of nerve roots at the corneoscleral limbus is 0.43–0.56 roots/mm (total number of nerve roots/πd). Based on the distribution patterns of canine corneal nerves, the nerve fibers of corneal nerve roots of canines emanated from the corneoscleral limbus to the central cornea. When a transparent corneal incision is made, the incision will be made on the cornea within about 1 mm to the corneoscleral limbus in a direction perpendicular to the radius of the cornea. The corneal nerves in the corresponding area could be cut off properly, which will directly damage the nerve roots at the corneoscleral limbus and the corneal nerve terminals, including the nerve branches between epithelial cells in the superficial layer and the nerve plexus in the corneal stromal layer. Even the overlapping branches extending from other regions of the corneal nerve would also be cut off [30]. In contrast, a scleral tunnel incision will be made parallel to the corneoscleral limbus from approximately 2.5 mm posterior to the corneoscleral limbus to a depth of 1/2 scleral thickness. The scleral tunnel knife will be adopted to peel forward along the scleral arc to the posterior corneoscleral limbus. Subsequently, the knife will be slightly elevated to avoid the location of the nerve roots at the corneoscleral limbus and peel forward along the corneal arc to 1 mm within the transparent cornea. Then, the puncture knife will be employed to puncture the anterior chamber. The depth of the tunnel knife in the cornea is approximately flush with the position of the corneal stromal layer. At this point, the dense nerve plexus within the stromal layer of the cornea will be partially cut off, while the nerve plexus in the superficial stromal layer will not be cut off. Besides, there are still nerve terminals from other zones of the cornea distributed in the corneal counterpart with the scleral tunnel incision. Therefore, it can be observed clinically that although the postoperative corneal sensation is diminished, it is not as significant as in the transparent corneal incision [18].

Currently, the incision length for the implantation of the soft intraocular lens is about 3.2 mm, and that the implantation of the rigid intraocular lens is about 5.5 mm in canine clinical cataract surgery. As per the density of nerve roots at the corneoscleral limbus (0.43–0.56 roots/mm), an average of 1.38–1.79 roots and 2.37–3.08 roots would be cut off, respectively, in these two common surgical incisions. Although the number of nerve roots cut for these two incisions is not large compared with the total number in the cornea (24.43), corneal nerves would intersect and anastomose with each other, which can partially compensate the cut region of nerve roots. However, even if the injury degree is small, more methods shall be taken to further reduce the injury degree of corneal nerves caused by corneal penetrating incision.

**Figure 3:**
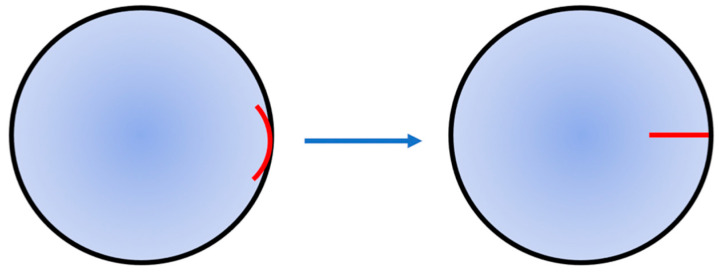
Schematic diagram of the recommended incision direction in corneal penetration surgery. The black line is the corneoscleral limbus. The red line is the surgical incision.

As revealed by the results of this study, the nerve roots in all zones of the canine cornea are evenly and symmetrically distributed. Therefore, there is no obvious difference in the injury degree, no matter which zone of the corneoscleral limbus is selected as the incision position. However, different incision directions would directly affect the injury degree of nerve roots. It can be maintained that the injury degree of nerve roots can be reduced to the lowest when the incision is made along the corneal radius. In the surgery that does not require the direction of corneal incision, the surgical approach of corneal penetrating surgery is adjusted from the direction perpendicular to radius to that along the radius (Figure 3), which can avoid the position of nerve roots at the corneoscleral limbus and reduce the injury degree of corneal nerves caused by corneal penetrating surgery. Although the incision along the radius may cut off the nerve fiber branches inside the cornea, it can protect the nerve roots at the corneoscleral limbus and minimize the injury degree of corneal nerves. Therefore, in order to reduce the postoperative complications caused by excessive transection of corneal nerves, it is a new idea worth exploring to adjust the incision direction of corneal penetrating surgery.

## 5. Conclusions

The corneal nerves of dogs emanated from the corneoscleral limbus in the direction of the corneal radius towards the center of the cornea, and gradually branched and intersected after entering the cornea, forming a corneal nerve network at different levels of the cornea. The nerve roots entering the cornea from the corneoscleral limbus are symmetrically distributed, and the nerve roots in the OS and OD are also symmetrically distributed. Besides, the number of corneal nerve roots had no correlation with the gender and age of canines. The adjustment of the surgical approach in the corneal penetrating surgery from the direction perpendicular to the radius to that along the radius can reduce the injury degree of corneal nerve roots caused by corneal penetrating surgery.

## Figures and Tables

**Table 1:** Total number of corneoscleral limbus nerve roots.

	No. Nerve Roots	SEM
Mean value	24.43	0.4623
95% Confidence interval of mean	23.43–25.42	
5% Revised average value	24.29	
Median	23.60	
Variance	9.45	
SD	3.0746	

SEM, Standard Error of Mean; SD, Standard deviation.

**Table 2:** Number of corneoscleral limbus nerve roots in male and female canines.

	Male		Female	
	No. Nerve Roots	SEM	No. Nerve Roots	SEM
Mean value	24.94	0.70	23.82	0.68
95% Confidence interval of mean	23.49–26.40		22.38–25.26	
5% Revised average value	24.69		23.80	
Median	24.60		23.20	
Variance	10.21		8.39	
SD	3.20		2.90	

SEM, Standard Error of Mean; SD, Standard deviation.

**Table 3:** Significant analysis of the number of nerve roots at the different zones of corneoscleral limbus in male and female canines.

	Type ⅢSS	DF	Mean Square	F-Value	*p*-Value
Corneal zones	9.371	5	1.874	1.993	0.081
Gender	2.029	1	2.029	2.157	0.143
SE	213.443	227	0.940		
Total	224.843	233			

SS, sum of square; DF, degree of freedom; SE, standard error.

**Table 4:** Number of corneoscleral limbus nerve roots in oculus sinister and oculus dexter.

	OS		OD	
	No. Nerve Roots	SEM	No. Nerve Roots	SEM
Mean value	24.56	0.66	24.27	0.76
95% Confidence interval of mean	23.19–25.93		22.66–25.87	
5% Revised average value	24.62		23.94	
Median	24.60		23.40	
Variance	9.05		10.44	
SD	3.01		3.23	

OS, Oculus Sinister; OD, Oculus Dexter; SEM, Standard Error of Mean; SD, Standard deviation.

**Table 5:** Significant analysis of the number of nerve roots at the different zones of corneoscleral limbus of oculus sinister and oculus dexter.

	Type ⅢSS	DF	Mean Square	F-Value	*p*-Value
Corneal zones	9.371	5	1.874	1.993	0.081
OS and OD	0.141	1	0.141	0.148	0.700
SE	215.331	227	0.949		
Total	224.843	233			

SS, sum of square; DF, degree of freedom; OS, Oculus Sinister; OD, Oculus Dexter; SE, standard error.

**Table 6:** Number of corneoscleral limbus nerve roots in different zones.

	Corneal Zones					
	a	b	c	d	e	f
Mean value	4.25	4.31	3.97	3.79	3.88	4.23
95 % Confidence interval of mean	3.86–4.64	4.06–4.56	3.68–4.27	3.58–4.00	3.49–4.26	33.90–4.55
5% Revised average value	4.26	4.30	3.94	3.74	3.86	4.22
Median	4.20	4.20	3.80	3.60	3.80	4.40
Variance	1.44	0.58	0.82	0.41	1.40	1.01
SD	1.20	0.76	0.91	0.64	1.18	1.00

SD, Standard deviation.

**Table 7:** Significance analysis of the number of corneoscleral limbus nerve roots in each zone and other zones.

Corneal Zones	a		b		c		d		e		f	
	Mean Error	*p*-Value	Mean Error	*p*-Value	Mean Error	*p*-Value	Mean Error	*p*-Value	Mean Error	*p*-Value	Mean Error	*p*-Value
a			0.06	1.00	−0.28	0.80	−0.46	0.29	−0.37	0.53	−0.03	1.00
b	−0.06	1.00			−0.34	0.64	−0.52	0.17	−0.43	0.36	−0.08	1.00
c	0.28	0.80	0.34	0.64			−0.18	0.96	−0.09	1.00	0.25	0.86
d	0.46	0.29	0.52	0.17	0.18	0.96			0.09	1.00	0.44	0.36
e	0.37	0.53	0.43	0.36	0.09	1.00	−0.09	1.00			0.35	0.61
f	0.03	1.00	0.08	1.00	−0.25	0.86	−0.44	0.36	−0.35	0.61		

**Table 8:** Significance analysis of the number of nerve roots at the corneoscleral limbus in canines of different ages.

Ages	Mean Value	95 % Confidence Interval of Mean	F-Value	*p*-Value
<1 Year (puppies)	25.17	22.71–27.62	1.192	0.324
1–2 Years (adult canines)	25.25	23.02–27.48		
2–3 Years (adult canines)	23.07	20.14–25.99		

## Data Availability

The datasets supporting the conclusions of this article are included within the article.
